# The development and impact of adopting electronic health records in the United States: A brief overview and implications for nursing education

**DOI:** 10.1002/hcs2.21

**Published:** 2022-11-01

**Authors:** Song Ge, Yuting Song, Jiale Hu, Xianping Tang, Junxin Li, Linda Dune

**Affiliations:** ^1^ Department of Natural Sciences, College of Sciences and Technology University of Houston‐Downtown Houston Texas USA; ^2^ School of Nursing Qingdao University Qingdao Shandong China; ^3^ Department of Nurse Anesthesia, College of Health Professions Virginia Commonwealth University Richmond Virginia USA; ^4^ School of Nursing Xuzhou Medical University Xuzhou Jiangsu China; ^5^ School of Nursing Johns Hopkins University Baltimore Maryland USA

**Keywords:** electronic health records, electronic medical record, US healthcare, health information system, health informatics, Nursing education

**Abbreviations**
BSN: Bachelor of science in nursing
EMR: Electronic medical records
EHR: Electronic health records
HIMSS: Healthcare Information and Management Systems Society
HITECH: Health Information Technology for Economic and Clinical Health

## 1 DEVELOPMENT AND ADOPTION OF EHR IN THE UNITED STATES

At present, health‐care systems in the United States face enormous challenges in providing quality care, characterized by safe, effective, efficient, patient‐centered, timely, and equitable care while containing health‐care costs [1, 2]. To understand and address patients' increasingly complicated health‐care needs, we need safe access to quality information that is characterized by integrity, reliability, and accuracy [3], and establish mutually beneficial relationships among a multidisciplinary team of professionals [4]. Traditional paper‐based clinical workflow produces many issues such as illegible handwriting, inconvenient access, the possibility of computational prescribing errors, inadequate patient hand‐offs, and drug administration errors. These problems can lead to medical errors, omissions, and duplications and, ultimately, poor patient outcomes and compromised quality of care [2].

Electronic health records (EHR) is a major achievement in the health information technology [5]. It is deemed a promising solution to improve the interoperability of patients' information across health‐care settings and achieve a more cost‐effective, safer, and higher quality of care [3, 6]. Electronic medical records (EMR) is a different concept from EHR; thus, the two terms cannot be used interchangeably. The EMR is the official record produced by hospitals and other ambulatory settings that serves as the EHR's data source. EMR is a prerequisite for EHR [7]. EHR refers to systematic documentation of patients' health status and health care in a secured digital format [8]. It indicates that patients' health information can not only be stored but also be transmitted and accessed by authorized interdisciplinary professionals across health‐care settings in patients' health‐care continuum. In addition, authorized non‐health‐care professionals, including insurers, the government, and researchers can also have access to patients' health information as well.

With EHR, patients can have greater autonomy over their care, and clinicians may better understand patients' medical history and coordinate care with other interdisciplinary professionals with fewer barriers [2]. EHR can also provide data for a variety of other purposes such as providing data for research, population‐based interventions, and reporting quality‐related measures [9]. Thus, this technological innovation benefits not only patients but also healthcare providers, administrative officers, researchers, and professionals from a variety of disciplines [10].

**Figure 1:**
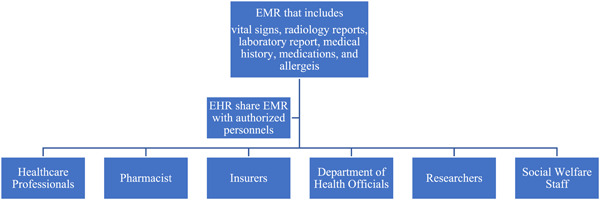
The basic flow of electronic health records (EHR)

The adoption of EHR in the United States started early and was accelerated by laws and regulations. In 2004, US President George W. Bush proposed a plan that most Americans would have EHR by 2014. He stated that computerizing health records could help clinicians avoid dangerous medical mistakes, reduce costs, and improve patients' care [11]. Later, President Obama continued this effort by proposing the American Recovery and Reinvestment Act of 2009 [12]. This policy included the Health Information Technology for Economic and Clinical Health (HITECH) Act to use Medicare and Medicaid to provide explicit reimbursement and penalties incentives for health organizations and providers to adopt EHR meaningfully within a specific time frame [10, 13]. Initiating this act, the federal government committed unprecedented resources to support the adoption of EHR [10]. The HITECH Act could be considered “the most significant driver” to encourage the adoption of EHR in the United States before the COVID‐19 pandemic [14]. It was important to note that not all EHR were eligible for reimbursement. The HITECH act specifies that health‐care providers and organizations must implement all the EHR's core objectives before selecting five of ten additional ones to accomplish during the first 2 years to be eligible for reimbursement. The fundamental tasks that supported better health care were included in the key objectives defined by the HITECH act and included the data entry and many software‐based clinical decision support systems (DSS) [10]. The optional objectives gave providers the opportunity to make choices based on their circumstances. A wide spectrum of health‐care companies in the United States has implemented EHR after more than 10 years, despite the fact that the technology and standards are constantly changing and there are still acceptance barriers. [15]. In 2001, only 18% of physicians used EHR, compared with over 80% in 2016 [16]. Moreover, according to the Healthcare Information and Management Systems Society (HIMSS) Analytics 2015 Report, 1313 US hospitals have achieved fully implementation of physician documentation, robust clinical DSS, and electronic access to medical imaging (Stage 6) [17] (Figure 1).

The coronavirus disease 2019 (COVID‐19) pandemic that has occurred since 2020 has had an unprecedented impact on the adoption of EHR in the United States. As EHR offered convenience, safety, quicker results reporting, and virtual visits, EHR was highly demanded during COVID‐19 when people's life was disrupted. In addition, with the order of President Trump, under the Stafford Act and the National Emergencies Act, the Center for Medicare and Medicaid temporarily expanded coverage for telehealth and virtual care visits [18]. Certain regulatory changes also took place, such as allowing providers to practice across state lines. All these pandemic‐related changes led to leaps in the adoption of EHR. The change was unprecedented. For example, following March 13, 2020, Intermountain Health saw a rise in telehealth visits from about 100 per month to over 50,000 per week [19]. Overall, COVID‐19 caused extensive short‐term and long‐term changes in people's attitudes toward as well as demand for HER.

## 2 COMPONENTS OF EHR (TABLE 1)

The detailed components of EHR are shown in Table 1.

**Table 1:** Components of EHR

Component	Definition
Health information and data	EHR contains crucial patient information required for clinical decision‐making, including patients' demographics, medical and nursing diagnoses, medication lists, allergy, medical history, test results, etc. The information could be used during patient registration, admission, transfer, and discharge.
Results management	EHR retains patients' comprehensive results reports of various types, including current and historical written, laboratory, and radiology reports.
Order entry management	Healthcare professionals from all disciplines should be able to enter various types of orders via EHR, including medication, laboratory, radiology, pathology, nutrition, supply, nursing, consultations orders, etc.
Decision support	The incorporation of clinical decision‐making support tools including drug–drug, drug–allergy, and drug–diagnosis interactions. By sending notifications and reminders to patients and providers, these solutions assist in error‐checking processes and boost the quality of care.
Electronic communication and connectivity	EHR encourage communication via email, web messaging, and other channels between healthcare teams, their members, and patients.
Patient support	By offering educational materials, telehealth, and home monitoring, EHR encourages patient education.
Administrative processes and reporting	EHR automatically records all fees related to patients' stays.
	EHR also enables systems for computerized scheduling, billing, and claims management.
Population health management	EHR supports standardized terminology, disease reporting, and clinical research, therefore promoting population health assessment, intervention, and evaluation.

Abbreviation: EHR, electronic health records.

## 3 CONCERNS AND BARRIERS TO ADOPTING EHR

1. Shift focus away from patients. According to a Delphi study, physicians expressed concerns that EHR would reduce the number of patients they were able to see in a day. In addition, they were worried that EHR would shift their focus away from patients to screens and may cause them to miss important medical information [20]. In another study, researchers supported this claim and found that physicians' screen gaze and keyboarding time were negatively correlated with patient‐centered communications during patients' visits. Specifically, screen gaze disrupted physicians' communication with patients by inhibiting eye contact, psychosocial inquiry, and emotional responsiveness between providers and patients [21]. Thus, more education is needed for physicians to understand how to optimize the use of EHR and achieve optimal interpersonal exchange with patients.
2. Distinctive interoperability of EHR and state laws and practices. Interoperability refers to the ability to exchange information among different information systems. The diversity of EHR vendors in the market makes exchanging information challenging. Moreover, different states have specific privacy laws concerning private data and various practices regarding health information technology, making interoperability across states challenging [13].
3. High cost of installing and maintaining EHR. EHR is expensive as the cost associated with adopting EHR is not a one‐time fee. Before adopting an EHR system, organizations must identify and assign appropriate administrative and medical personnel to work on implementation with a tight network with EHR vendors. The other costs include hardware (computers, laptops, all‐in‐one computers, tablets, mouses, keyboards, monitors, and supporting tables), EHR software which could vary a lot based on the type of EHR, IT support, training in how to use EHR, loss of productivity during installation, and ongoing network fees and maintenance [22, 23]. The associated expenses could be challenging, especially for small healthcare agencies in remote areas with limited resources.
4. Human resistance. According to a survey, providers may not be willing to take the time to familiarize themselves with the available systems, select the suitable EHR, implement it, or get trained to use it. In addition, providers may not possess adequate computer skills to exert all the functions of EHR and deem EHR too complicated to use [24]. Last but not least, providers have concerns that EHR generates new issues on patients' privacy and confidentiality. All these reasons hamper their desire to use EHR [25].
5. Design issues of EHR. Poor design and use of EHR can lead to errors, adverse events, and even mortality [26]. This is further aggravated by the increasing functional complexities of EHR to meet clinicians' demands and the pressure to adopt them within a specific time frame [3, 27]. In one study, researchers identified many EHR‐related errors resulting from poorly designed EHR that could threaten patients' safety, compromise the quality of care, and lead to abuse and fraud of health information [3]. These errors include poor system usabilities, such as confusing interfaces and workflow incompatibility, over‐alerts that lead to clinicians' fatigue and ignorance, inappropriate copy and paste of information, and mistakes in documentation capture. With EHR, there are also greater risks of breaking down information integrity with hackers, malicious insiders, and passcode leakage. In one study, researchers noticed an unexpected increase in death after implementing a computerized physician order entry system in a children's hospital [28]. These challenges require efforts from both EHR developers and providers to overcome by designing and implementing solid usability standards to optimize system safety to achieve information integrity [27].

## 4 THE POSITIVE IMPACT OF EHR ON US HEALTHCARE

1. Improved financial outcome. Studies suggested that physicians using EHR had improved clinical care, practice efficiency, and finances than those nonadopters [29]. Another cost–benefit study demonstrated that in the United States, EHR helped save an average of USD 86,400 per provider over 5 years in the primary care setting by reducing drug expenditures, improving utilization of radiology tests, better‐capturing charges, and decreasing billing errors [30].
2. Improved quality of care. EHR provides providers with information in a way that paper formats cannot achieve. Providers could see instant information, view and track trends of values, and receive alerts and suggestions for decision‐making [4]. In a study conducted in an ambulatory care setting, EHR use was associated with a higher quality of care on hemoglobin A1c testing for diabetic patients, breast cancer screening, chlamydia screening, and colorectal cancer screening, with an effect size ranging from 3% to 13% per measure [31]. In another study on patients' perceptions, patients who reported physician use of EMRs had higher self‐reported care quality after controlling for sociodemographic characteristics, usual source of health care, and health insurance status [32]. According to a national survey, 82% of physicians who used EHR reported improved clinical decision‐making, 92% reported improved communication with other providers and their patients, and 82% reported reduced medication errors [33]. Then, in another study, researchers suggested that the benefits of EHR implementation on improved efficiency exceeded the costs of adoption [34]. In addition, according to a systematic review and meta‐analysis, EHR use was associated with reduced documentation time, higher guideline adherence for physicians, and more minor medication errors and adverse drug effects for patients [35]. Assimilation of EHR at a hospital‐wide level is probably more critical than pure adoption. A study demonstrated that the former could improve the efficiency of patients by reducing the length of patients' hospital stays, especially for those with greater health complexities [36].
3. Improved patient satisfaction. In a systematic review of eight included studies evaluating patient satisfaction with EHR, researchers noted a significant improvement in patients' satisfaction after providers implemented EHR. However, many studies have quality issues such as low response rate, no definition of patients' satisfaction, and lack of validity and reliability in their measurement. Thus, the extent to which the conclusion is valid is limited.
4. Saved documentation time. In a study comparing nurses' documentation time before and after implementing an EHR on a medical‐surgical nursing unit [37], researchers found that after the implementation of computerized physician order entry, documentation time for nurses was reduced. In another study [38], nurses reported that they could finish their work much faster after the implementation of an Emergency Department EMR.

## 5 DISCUSSIONS

Using EHR to improve healthcare has been a strategy that raises many countries' attention and efforts because of EHR's vast potential and functionalities. However, this is never an easy process and can be viewed as a revolution due to its complexities and scope of change. In this review, we identified several barriers to its adoption from financial, technical, and human aspects. Healthcare institutions should carefully attend to these considerations if they plan to adopt EHR. Rushing this process does not help implement such a large‐scale campaign and could lead to worse rather than better outcomes, as illustrated by many earlier studies mentioned above. In particular, hospitals in many developing countries are at an early stage of EHR adoption [39] with a tremendous amount of work that needs to be done. Legislation should be in place regarding guidelines and standards for EHR that are allowed to be implemented with particular attention to interoperability among distinctive. Making EHR systems become interoperability is a widespread challenge. Legislation could facilitate the adoption of EHR by providing health‐care agencies with incentives and facilitators to implement specific instructions.

Meanwhile, health‐care agencies should develop an appropriate timeline to adopt EHR, including (a) Identifying the information needs of their organization, (b) understanding the current market of EHR market, and (c) assigning interdisciplinary expertise to choose the desired system among a vast potential selection of vendors and systems, (d) carefully examining features of EHR, (e) getting the hardware ready, (f) adjusting the EHR to fit the need of their specific organization, (g) carefully train personnel, (h) decreasing users' resistance by providing robust and ongoing support, and (i) maintaining and updating the system at regular basis. It is also essential to develop mechanisms to evaluate the impact of EHR on healthcare professionals' workflow efficiency, quality of care, and patient outcomes so that any mistake and weakness can be caught early. Input from multidisciplinary teams is valuable and needed because each profession will bring unique perspectives and have special needs for EMR functions. Overall, we not only need adoption but more integration of EHR within the daily workflow of healthcare agencies and production of better patient outcomes.

## 6 IMPLICATIONS FOR PROFESSIONAL NURSING DEVELOPMENT

The wide disparity in nurses' informatics competence has negatively affected their utilization of EHR [40]. Nurses need to be willing to learn the strengths and features of EHR over the traditional paper approach and constantly improve their informatics competence to adapt to the changing technology such as big data, artificial intelligence, robotics, and telehealth. This is particularly important during the COVID‐19 pandemic when remote diagnosis is expanding quickly. Nurses' EHR learning process can start early to achieve the best results. Nursing students should develop informatics competence in their education. Nursing educators should be aware that fostering a favorable attitude toward using EHR and elevating the perceived value in their nursing students is crucial for improving their acceptance of using them [41]. However, nursing educators from academic institutions are frequently left out of the deployment of EHR themselves and nursing schools often lack EHR education resources [42]. Moreover, a consensus is lacking on the content of information education for bachelor of science in nursing (BSN) students [43]. Thus, the integration of informatics into BSN education has been relatively slow [40]. Many new graduate nurses were not healthcare informatics competent [42].

Nursing educators must develop effective strategies to incorporate informatics into nursing education and make the education content pragmatic, relevant, and appealing to nursing students. Important concepts should be included in the curriculum, such as the development of EHR, its impact on the health‐care system, examples of technology and information systems that are effective and safe within various practice settings, and how to safeguard patients' information. In addition, researchers also found that a simulated EHR curriculum is an effective and engaging approach to teaching students EHR skills and organizing charts leading to a safe, effective, and high‐quality patient care [44]. In a simulated EHR curriculum, students draft orders and prescriptions using an EHR training platform, develop an evidence‐based nursing care plan, and conduct a small‐group review of their work after viewing a virtual medical record of a complex patient with chronic conditions and compromised care.

Nurses should be competent to use EHR at workplaces after a rigorous selection of the appropriate EHR system in their health‐care agency and relevant support provided. As the frontier of healthcare, nurses have great opportunities to participate in this significant revolution. Nurses could work during the preinstallation phase such as helping the agency choose the most suitable system, adjusting the system to the need of their agency with their expertise, encouraging and training their colleagues for adoption, and assisting their agency in evaluating the quality, adoption, and impact of the system. Ultimately, with everyone's efforts, the system will provide all health‐care professionals, including nurses better working processes and care outcomes for patients if integrated well with the agency.

## AUTHOR CONTRIBUTIONS

**Song Ge**: Conceptualization (equal); Investigation (equal); Methodology (equal). **Yuting Song**: Conceptualization (equal); Resources (equal). **Jiale Hu**: Resources (equal); Software (equal); Supervision (equal). **Xianping Tang**: Investigation (equal); Software (equal); Supervision (equal). **Junxin Li**: Conceptualization (equal); Data curation (equal); Formal analysis (equal); Writing – original draft (equal); Writing – review & editing (equal). **Linda Dune**: Formal analysis (equal); Funding acquisition (equal).

## CONFLICT OF INTEREST

The authors declare no conflict of interest.

## ETHICS STATEMENT

Not Applicable.

## INFORMED CONSENT

Not Applicable.

## Data Availability

Data sharing not applicable to this article as no datasets were generated or analyzed during the current study.
